# Effect of core stabilization exercises on antioxidant/oxidant levels in hypertensives

**DOI:** 10.1590/1806-9282.20250600

**Published:** 2025-12-05

**Authors:** Serkan Duyuler, Hale Saribas, Melike Sura Oksuz Capanoglu, Ulas Ar, Ebru Calik Kutukcu, Pinar Turker Duyuler

**Affiliations:** 1 Bilkent City Hospital – Ankara, Turkey.; 2 Ardahan State Hospital – Ardahan, Turkey.; 3 Hacettepe University, Faculty of Physical Therapy and Rehabilitation – Ankara, Turkey.; 4 Bilkent City Hospital, Department of Cardiology – Ankara, Turkey.

**Keywords:** Hypertension, Exercise, Core stability, Oxidative stress, Asymmetric dimethylarginine, Antioxidant

## Abstract

**OBJECTIVE:**

The aims of this study were to investigate the effects of core stabilization exercises delivered through telerehabilitation on blood pressure, quality of life, and levels of asymmetric dimethylarginine, total antioxidant status, total oxidant status, and oxidative stress index in hypertensive patients.

**METHODS:**

We recruited 58 individuals diagnosed with hypertension and randomized 29 patients to the core stabilization exercise group and 29 patients to the control group. Patients in the exercise group performed core stabilization exercises under supervision of a physiotherapist by telerehabilitation. The measurements of asymmetric dimethylarginine, total antioxidant status, total oxidant status, oxidative stress index, ambulatory blood pressure, and questionnaires were evaluated before and after the 12-week training.

**RESULTS:**

Compared to the basal levels, total antioxidant status (1.848±0.207 vs. 1.861±0.198; p=0.784), total oxidant status (17.872±5.552 vs. 19.288±5.516; p=0.165), oxidative stress index (9.770±3.073 vs. 10.450±3.113; p=0.252), and asymmetric dimethylarginine (825.2±165.1 vs. 817.4±161.6; p=0.718) did not differ after the 12 weeks in control group. After 12-week training, total antioxidant status (1.814±0.232 vs. 1.936±0.294; p=0.010), total oxidant status (17.798±6.799 vs. 15.492±4.157; p=0.022), oxidative stress index (9.888±3.683 vs. 8.099±2.107; p=0.009), and asymmetric dimethylarginine (848.6±143.7 vs. 807.1±127.3; p=0.010) were improved in exercise group compared to basal levels. Quality of life, anxiety, and fatigue severity levels did not differ in both groups after 12 weeks. The sleep quality was slightly improved in both groups after 12 weeks (exercise group: 8.2±2.9 vs. 7.2±2.5; p>0.001 and control group: 8.6±4.2 vs. 7.6±3.9; p=0.007).

**CONCLUSION:**

Core stabilization exercises may improve oxidative balance and endothelial function in patients with hypertension.

## INTRODUCTION

Emerging evidence suggests that core stabilization (CS) exercises may exert beneficial effects on systemic oxidative stress by lowering total oxidant status (TOS)^1^. However, scientific data on their specific impact on vascular health and cardiovascular outcomes remain limited and warrant further investigation^2^. An imbalance between oxidative and antioxidative mechanisms is a key contributor to endothelial dysfunction in patients with hypertension (HTN)^3^. One important mediator in this process is asymmetric dimethylarginine (ADMA), an endogenous inhibitor of nitric oxide synthase (NOS). Elevated ADMA levels impair NOS activity, resulting in decreased nitric oxide (NO) bioavailability. As NO is a critical endothelium-derived vasodilator with protective roles in maintaining vascular tone and structure, its deficiency leads to hemodynamic alterations and promotes endothelial dysfunction. Accumulating evidence indicates that ADMA plays a central role in the pathogenesis of HTN^4^.

Telemedicine and telerehabilitation enhance self-management and care access, improving quality of life, reducing hospitalizations, enabling earlier discharge, and offering cost-effective, timely rehabilitation—especially for underserved populations^5,6^.

Evidence related to the effectiveness of CS training in patients with HTN is limited. A study reported a significant increase in heart rate (HR), and systolic and diastolic blood pressure in healthy subjects with CS training^7^. Low-intensity core training like Pilates improves antioxidant activity, reduces oxidative and inflammatory markers, and boosts NO, indicating potential systemic benefits on oxidative stress in hypertensive patients^8^. Although core stabilization exercises via telerehabilitation have been studied in other populations, evidence specific to hypertensive patients remains limited^9,10^. Therefore, we aimed to investigate the effects of CS exercises delivered by telerehabilitation on blood pressure, endothelial function, antioxidant/oxidant levels, sleep quality, anxiety, fatigue severity, and quality of life in hypertensive patients in this study.

## METHODS

### Patients

We performed an a priori analysis using G*Power. Assuming a moderate effect size (Cohen's d=0.55), α=0.05, and power=0.80, the required sample size for a paired samples t-test was estimated to be 27 participants per group. Since the current study included 29 participants per group, the sample size can be considered adequate to detect moderate within-group changes. We recruited 58 individuals diagnosed with stage I - II HTN and HTN treatments implemented according to the current treatment guidelines, who were evaluated with an ambulatory blood pressure monitor (Mobil-O-Graph, IEM GbH, Aachen, Germany) between December 2021 and June 2022. Antihypertensive treatments were personalized and administered per current guidelines to help patients reach target blood pressure, ensuring optimal, individualized care during the study. The eligibility criteria for participation in the study are defined as follows: Inclusion Criteria: (1) Clinically stable, (2) Age 25–70 years, (3) Voluntary participation, (4) Literate, and (5) Able to use a smartphone, tablet, or computer for online programs. Exclusion Criteria: (1) Unstable conditions (decompensated heart failure, unstable angina, severe valve disease, and chronic kidney failure), (2) Severe neuromuscular or musculoskeletal disorders, (3) Unable to complete questionnaires, (4) Pregnant or diagnosed with chronic respiratory disease, and (5) Unwilling to participate.

We randomized 29 patients to the core stabilization exercise group (EG) and 29 patients to the control group (CG). Following the randomization procedure, the participants in the EG performed CS exercises via video calls with a physiotherapist in an online environment. The exercise difficulty increased progressively throughout the 12-week program to match individual capabilities and was modified as needed. Each session lasted 45 min, twice a week. The CS exercises were planned to focus on increasing participants’ strength of core muscles, and session attendance was >90% and was monitored via real-time video supervision. Patients underwent assessments before the program. The baseline measurements included demographic and clinical characteristics, biochemical analyses, serum ADMA levels, TOS, total antioxidant status (TAS), oxidative stress index (OSI), echocardiography evaluation, and questionnaires. Ambulatory blood pressure recordings were obtained before the program. The serum ADMA, TAS, TOS, OSI, ambulatory blood pressure, and questionnaires were reevaluated after the 12-week program. No participants dropped out, resulting in complete data for all randomized individuals; therefore, the intention-to-treat analysis was not required.

Blood samples were drawn from the antecubital vein after a 12-h fast. Serum ADMA levels were measured via ELISA, while TAS and TOS were analyzed using commercial kits (Rel Assay Diagnostics, Turkey) with an autoanalyzer per Erel's method. OSI was calculated as the TOS/TAS ratio^11^.

The Turkish version of the short form (SF-36) was used in the assessment of quality of life and general health profile^12^. The Pittsburgh Sleep Quality Index (PSQI) was used to assess sleep quality^13^. The State-Trait Anxiety Inventory (STAI 1–2) was used to measure participants’ anxiety levels^14^. The Fatigue Severity Scale (FSS) was used to assess fatigue perception degree during daily life^15^.

The study adhered to the principles specified in the Helsinki Declaration for biomedical research involving human subjects. The local Ethical Committee of (Bilkent City Hospital) approved the study (Approval number: E2-21-589). The written informed consent was provided for each participant.

### Statistical analysis

Statistical analyses were conducted using SPSS v18 (SPSS Inc, Chicago, USA). Normality was assessed with the Shapiro-Wilk test. Non-normally distributed variables were analyzed with the Mann-Whitney U test, while parametric tests were applied for normally distributed data. Paired t-tests assessed pre- and post-intervention differences, and independent t-tests compared groups. Data were reported as mean±SD (normal), median [25th–75th percentile] (non-normal), and n (%; categorical). Statistical significance was set at p<0.05.

## RESULTS

Fifty-eight (30 female and 28 male) patients with a mean age of 48.9±8.2 years were included in the study. Twenty-nine patients were in the CG, and 29 patients were in the EG. Table 1 shows the demographic and clinical characteristics of the patients. Age, frequency of diabetes mellitus, and smoking were similar in both groups (p=0.277, p=1.0, and p=0.431, respectively). Also, basal biochemical and echocardiographic parameters did not differ significantly between groups. Number of antihypertensive medications used for blood pressure was significantly higher in the CG [2 (1–3) vs. 1 (1–2); p=0.008].

**Table 1:** Demographic, clinical, and laboratory characteristics of the study population.

		Control group (n=29)	Exercise group (n=29)	p
Age, years		50.1±8.1	47.7±8.4	0.277
Sex, female (%)		16 (55.2)	14 (48.3)	0.599
Smokers (%)		12 (41.4)	16 (55.2)	0.431
Diabetes mellitus (%)		6 (20.7)	5 (17.2)	1.000
Creatinine, mg/dL		0.83±0.2	0.83±0.14	0.933
Urea, mg/dL		31.0±6.1	30.8±9.5	0.934
Total cholesterol, mg/dL		209.0±34.9	212.1±38.5	0.754
Triglyceride, mg/dL		142 (100.5–216)	165 (117.5–218)	0.663
High density lipoprotein, mg/dL		49.4±15.4	48.2±10.7	0.759
Low density lipoprotein, mg/dL		125.3±30.7	130.9±35.5	0.519
Thyroid-stimulating hormone, mU/l		1.97 (1.03–2.96)	1.48 (1–2.32)	0.079
Glucose, mg/dL		107.3±48.9	101.1±32.4	0.573
Leukocytes, /mm^a^		7,435±1,723	7,759±1,780	0.485
Hemoglobin, g/dL		14.2±1.30	14.7±1.7	0.259
Platelets, /mm^a^		268,655±48,740	272,103±60,849	0.813
C-reactive protein, mg/l		1 (0–2)	2 (0–3.5)	0.091
Total antihypertensive medications		2 (1–3)	1 (1–2)	0.008
Total antioxidant status, total oxidant status, and asymmetric dimethylarginine levels				
	Initial TAS, mmol Trolox Eq/L	1.848±0.207	1.814±0.232	0.557
	Final TAS, mmol Trolox Eq/L	1.861±0.198	1.936±0.294	0.263
	Initial TOS, **μ**mol H2O2 Eq/L	17.872±5.552	17.798±6.799	0.964
	Final TOS, **μ**mol H2O2 Eq/L	19.288±5.516	15.491±4.157	0.005
	Initial OSI, arbitrary unit	9.770±3.0734	9.888±3.683	0.895
	Final OSI, arbitrary unit	10.450±3.114	8.099±2.107	0.001
	Initial ADMA, ng/mL	825.2±165.1	848.6±143.7	0.567
	Final ADMA, ng/mL	817.4±161.6	807.1±127.3	0.789

ADMA: asymmetric dimethylarginine; TAS: total antioxidant status; TOS: total oxidant status; OSI: oxidative stress index.

Basal TAS (1.848±0.207 vs. 1.814±0.232; p=0.557), TOS (17.872±5.552 vs. 17.798±6.799; p=0.964), OSI (9.770±3.0734 vs. 9.888±3.683; p=0.895), and serum ADMA levels (825.2±165.1 vs. 848.6±143.7; p=0.567) were similar in both groups. In the twelfth-week evaluation, TAS (1.861±0.198 vs. 1.936±0.294; p=0.263) and serum ADMA levels (817.4±161.6 vs. 807.1±127.3; p=0.789) were comparable in both groups; on the other hand, TOS (19.288±5.516 vs. 15.491±4.157; p=0.005) and OSI (10.450±3.114 vs. 8.099±2.107; p=0.001) were significantly lower in the EG.

Compared to the basal levels, TAS (1.848±0.207 vs. 1.861±0.198; p=0.784), TOS (17.872±5.552 vs. 19.288±5.516; p=0.165), OSI (9.770±3.073 vs. 10.450±3.113; p=0.252), and ADMA (825.2±165.1 vs. 817.4±161.6; p=0.718) levels did not differ after the 12 weeks in the CG. After the 12-week exercise period, TAS (1.814±0.232 vs. 1.936±0.294; p=0.010), TOS (17.798±6.799 vs. 15.492±4.157; p=0.022), OSI (9.888±3.683 vs. 8.099±2.107; p=0.009), and serum ADMA (848.6±143.7 vs. 807.1±127.3; p=0.010) levels were improved in the EG when compared to basal levels (see Table 2). After applying Benjamini-Hochberg FDR correction for multiple comparisons among oxidative stress and endothelial markers (TAS, TOS, OSI, and ADMA), all adjusted p-values remained statistically significant (p<0.05), supporting the robustness of the observed effects. Specifically, the adjusted p-values were: OSI=0.036, TAS=0.040, ADMA=0.040, and TOS=0.044. Additionally, blood pressures were significantly decreased in both control and EGs after the 12 weeks (Table 2).

**Table 2:** Comparison of initial and final levels of total antioxidant status, total oxidant status, serum asymmetric dimethylarginine, and ambulatory blood pressure monitoring measurements.

		Control group (n=29)			Exercise group (n=29)		
		Baseline	Final	p	Baseline	Final	P
TAS, mmol Trolox Eq/L		1.848±0.207	1.861±0.198	0.784	1.814±0.232	1.936±0.294	**0.010** *
TOS, **μ**mol H2O2 Eq/L		17.872±5.552	19.288±5.516	0.165	17.798±6.799	15.492±4.157	**0.022** *
OSI, arbitrary unit		9.770±3.073	10.450±3.113	0.252	9.888±3.683	8.099±2.107	**0.009** *
Serum ADMA, ng/mL		825.2±165.1	817.4±161.6	0.718	848.6±143.7	807.1±127.3	**0.010** *
Ambulatory blood pressure monitoring measurements							
	Total recording duration, h	23.5±0.8	24.0±1.7	0.094	23.8±1.1	23.8±0.7	1.000
	Daytime duration, h	14.5±0.8	15.0±1.7	0.094	14.8±1.1	14.7±0.8	0.899
	Nighttime duration, h	9±0	9±0	0.461	9±0	9±0.2	0.327
	Daytime measurement count	29.3±2.2	29.9±4	0.841	29.8±2.2	29.7±1.7	0.786
	Nighttime measurement count	8.8±0.5	8.9±0.9	**0.792**	9±0	9±0.3	0.574
	24-h average systolic blood pressure, mmHg	146.3±12.6	136.8±12.2	**0.001** *	143.3±8.1	132.7±10	**<0.001** *
	24-h average diastolic blood pressure, mmHg	90.4±7.4	85.4±7.9	**0.004** *	90.4±6.8	83.2±9.2	**<0.001** *
	Daytime average systolic blood pressure, mmHg	148.7±12.2	139.8±12.3	**0.003** *	145.9±8.2	135.0±9.5	**<0.001** *
	Daytime average diastolic blood pressure, mmHg	92.7±7.4	87.7±8.4	**0.002** *	92.7±7.0	85.0±9.2	**<0.001** *
	Nighttime average systolic blood pressure, mmHg	138.4±15.8	127.1±13.7	**0.021** *	134.8±13.4	125.7±13.9	**0.001** *
	Nighttime average diastolic blood pressure, mmHg	81.9±8.7	77.8±8.2	**0.004** *	82.9±9.1	77.1±10.8	**<0.001** *

ADMA: asymmetric dimethylarginine, TAS: total antioxidant status, TOS: total oxidant status, OSI: oxidative stress index.

* p<0.05.

The SF-36, Anxiety, and Fatigue severity did not differ between basal and twelfth-week evaluations in both groups (Table 3). Sleep quality was slightly improved in both groups after 12 weeks (CG: 8.6±4.2 vs. 7.6±3.9; p=0.007 and EG: 8.2±2.9 vs. 7.2±2.5; p>0.001). Basal exercise benefit and barrier scale scores were similar after the twelfth-week evaluation in the CG. In the EG, the exercise barrier scale score was slightly increased after 12 weeks (27.7±6.8 vs. 29.3±9.0; p=0.034).

**Table 3:** Scores of questionnaire in initial and final evaluations.

	Control group (n=29)			Exercise group (n=29)		
	Initial	Final	p	Initial	Final	P
Exercise barrier scale	30.6±7.5	30.3±6.6	0.794	27.7±6.8	29.3±9.0	0.034
Exercise benefit scale	58.6±14.3	56.9±14.1	0.551	55.3±12.4	54.5±11.7	0.094
PSQI total score	8.6±4.2	7.6±3.9	0.007	8.2±2.9	7.2±2.5	>0.001
SF-36 Physical functioning	69.7±24.0	73.4±23.4	0.228	83.6±15.1	77.6±21.8	0.068
SF-36 Role limitations due to physical health	76.1±26.1	82.7±21.1	0.301	84.0±21.5	72.0±32.5	0.056
SF-36 Role limitations due to emotional problems	70.5±30.9	72.6±29.4	0.837	83.3±27.6	75.9±30.4	0.280
SF-36 Energy/fatigue	50.4±23.2	58.2±21.6	0.089	58.0±19.7	58.4±22.6	0.911
SF-36 Emotional well-being	58.6±21.1	62.8±21.6	0.311	61.5±19.9	60.3±19.1	0.576
SF-36 Social functioning	65.6±20.3	71.3±21.5	0.072	74.0±21.9	77.5±21.0	0.430
SF-36 Pain	63.5±26.2	68±24.4	0.356	63.8±26.0	66.9±27.4	0.347
SF-36 General health	50.2±18.8	52.1±19.4	0.606	59.5±21.5	57.8±20.5	0.568
STAI State Anxiety Inventory	37.7±9.1	36.6±9.9	0.347	36.6±8.8	35.1±7.0	0.286
STAI Trait Anxiety Inventory	46.0±7.7	43.9±8.5	0.211	44.7±5.9	45.2±6.9	0.705
FSS score	4.5±1.8	4.3±1.8	0.635	3.6±1.6	4±1.9	0.136

SF: short form, STAI: State-Trait Anxiety Inventory, FSS: Fatigue Severity Scale, PSQI: Pittsburgh Sleep Quality Index.

## DISCUSSION

Core stabilization exercises via telerehabilitation may modestly improve oxidative and endothelial markers in hypertensive patients alongside standard therapy; however, no significant effect was observed on quality-of-life measures, limiting broader clinical implications.

Evidence indicates that oxidative stress plays a role in the pathophysiology of HTN. However, the effects of exercise on oxidative stress markers in hypertensive patients remain insufficiently explored. In a rat study, chronic exercise led to decreased TAS and increased TOS levels^16^. Conversely, a study in diabetic patients showed increased TAS without changes in TOS following chronic exercise, alongside reductions in blood pressure in the EG^17^. In our study, compared to baseline, we found that TAS levels were significantly higher and TOS levels were lower in the EG after 12-week exercise periods. This may indicate the possible favorable effect of exercise on oxidative stress in hypertensive patients.

Although the absolute changes in TAS and TOS were modest, their statistical significance and consistent directional shift suggest biological relevance—particularly in hypertensive individuals who experience chronic oxidative stress. The simultaneous increase in TAS, decrease in TOS, and reduction in OSI indicate a net improvement in redox balance. These findings may reflect potential long-term clinical benefits when integrated with lifestyle interventions such as exercise.

Asymmetric dimethylarginine serves as an indicator of endothelial dysfunction in individuals diagnosed with essential HTN. Previous studies showed that aerobic exercise training may decrease circulating ADMA levels in middle-aged and older adults^18^. However, the effect of exercise on ADMA levels in hypertensive patients has not been sufficiently investigated. In a study conducted in stage 1 hypertensive patients, intensive lifestyle treatment including diet and exercise reduced the ADMA levels compared to baseline^19^. Compared to our study, in the aforementioned study, patients reported their physical activity levels in metabolic equivalent rather than following a specific training program. In our study, we observed improvement in ADMA levels following a 12-week exercise program among participants in the EG. This finding suggests a beneficial effect of core stabilization exercise on endothelial functions among individuals with HTN. Although the observed reduction in ADMA levels in the EG was statistically significant, the absolute change (from 848.6 to 807.1 ng/mL) may be of limited clinical relevance. Given the lack of universally accepted reference ranges for circulating ADMA, it is difficult to determine a precise clinical threshold. However, prior studies have suggested that elevated levels (e.g., >500–600 ng/mL) are associated with endothelial dysfunction and increased cardiovascular risk. Therefore, while our findings may indicate a favorable physiological shift, their true clinical impact remains to be clarified in future longitudinal or outcome-based studies.

Our findings are consistent with previous research indicating that low-intensity, core-based exercise interventions can influence systemic oxidative balance. In a recent randomized controlled trial, it was found that hypertensive elderly patients who underwent 12 weeks of mat Pilates training experienced significant improvements in antioxidant enzyme activity and inflammatory marker reduction, alongside enhanced NO levels and blood pressure control^8^. Although Pilates and CSE differ in movement specificity, both share a focus on core muscle activation and low-to-moderate intensity. These results suggest a plausible mechanism by which CSE may contribute to systemic oxidative stress modulation through enhanced autonomic balance, improved vascular shear stress, and possibly endothelial NO production.

Hypertension negatively impacts quality of life, mental health, and sleep quality^20^. Prior studies show that physical activity can improve some quality-of-life domains in hypertensive patients, particularly physical function, general health, and vitality^21^. In contrast, our study found no significant changes in quality-of-life measures (SF-36), anxiety, or fatigue after 12 weeks of telerehabilitation-based core stabilization exercises in either group. This discrepancy may be due to differences in exercise modality and delivery; telerehabilitation's limited social interaction might reduce psychological benefits compared to group or face-to-face interventions.

Sleep quality was poor in both groups at baseline and showed improvement likely linked to antihypertensive treatment rather than exercise. This aligns with the literature suggesting that sleep disturbances contribute to HTN via increased sympathetic activity and endothelial dysfunction, while blood pressure control can in turn improve sleep^22^. These findings underscore the complex bidirectional relationship between HTN and sleep quality.

Although our study utilized composite oxidative stress markers (TAS and TOS), these reflect overall redox status and are influenced by multiple enzymatic and non-enzymatic systems. Prior studies on antioxidant agents like melatonin in animal models support the plausibility that modulating oxidative pathways can yield measurable improvements in oxidative stress^23^. Similarly, structured exercise programs have shown systemic benefits—such as improved cardiorespiratory function and body composition—in various populations, including post-COVID-19 patients^24^. Our findings, though specific to core stabilization exercises in hypertensive individuals, align with these broader physiological effects, highlighting the potential of targeted exercise interventions in chronic disease management.

### Study limitations

This study has several limitations. The absence of a healthy CG and short follow-up may limit generalizability. Although baseline characteristics and blood pressure medications were comparable, individualized adjustments may have introduced confounding. To enhance internal validity, only newly diagnosed hypertensive patients were included; sham or normotensive controls were not feasible due to ethical and resource constraints. Future studies should compare core stabilization exercises with other modalities such as aerobic or resistance training, both in-person and via telerehabilitation, to better elucidate their mechanisms and effects.

## CONCLUSION

Core stabilization exercises delivered via telerehabilitation, alongside appropriate medical treatment, may improve oxidative balance and serum markers of endothelial function in hypertensive patients.

## Data Availability

The datasets generated and/or analyzed during the current study are available from the corresponding author upon reasonable request.
